# Misperceiving Momentum: Computational Mechanisms of Biased Striatal Reward Prediction Errors in Bipolar Disorder

**DOI:** 10.1016/j.bpsgos.2024.100330

**Published:** 2024-04-30

**Authors:** Hestia Moningka, Liam Mason

**Affiliations:** a Research Department of Clinical, Educational and Health Psychology, University College London, London, United Kingdom; b Max Planck University College London Centre for Computational Psychiatry and Ageing Research, University College London, London, United Kingdom; c Wellcome Trust Centre for Human Neuroimaging, University College London, London, United Kingdom

**Keywords:** Affect, Bipolar disorder, Dopamine, Mood, Reward, Risk taking

## Abstract

**Background:**

Dysregulated reward processing and mood instability are core features of bipolar disorder that have largely been considered separately, with contradictory findings. We sought to test a mechanistic account that emphasizes an excessive tendency in bipolar disorder to enter recursive cycles in which reward perception is biased by signals that the environment may be changing for the better or worse.

**Methods:**

Participants completed a probabilistic reward task with functional magnetic resonance imaging. Using an influential computational model, we ascertained whether participants with bipolar disorder (*n* = 21) showed greater striatal tracking of momentum-biased reward prediction errors (RPEs) than matched control participants (*n* = 21). We conducted psychophysiological interaction analyses to quantify the degree to which each group modulated functional connectivity between the ventral striatum and left anterior insula in response to fluctuations in momentum.

**Results:**

In participants with bipolar disorder, but not control participants, the momentum-biased RPE model accounted for significant additional variance in striatal activity beyond a standard model of veridical RPEs. Compared with control participants, participants with bipolar disorder exhibited lower insular-striatal functional connectivity modulated by momentum-biased RPEs, an effect that was more pronounced as a function of current manic symptoms.

**Conclusions:**

Consistent with existing theory, we found evidence that bipolar disorder is associated with a tendency for momentum to excessively bias striatal tracking of RPEs. We identified impaired insular-striatal connectivity as a possible locus for this propensity. We argue that computational psychiatric approaches that examine momentary shifts in reward and mood dynamics have strong potential for yielding new mechanistic insights and intervention targets.

## Plain Language Summary

Bipolar disorder is characterized by extremes in mood and dysregulated reward processing. Moningka and Mason evaluated a neurocomputational model in which mood disturbances arise from an excessive tendency for momentum over recent experiences to bias reward perception. Using model-based functional MRI, the authors found that in contrast to matched control participants, participants with bipolar disorder exhibited fluctuations in reward-related neural responses that are modulated by momentum. They discuss this excessive neural tracking of momentum as one of the mechanisms that may underlie the propensity to enter recursive mood cycles in bipolar disorder.

Mood instability is emerging as an important feature across psychiatric disorders including mood disorders, psychotic disorders, and personality disorders (1,2). It is characterized as a tendency to experience frequent oscillations of intense affect to a degree that impacts one’s ability to regulate these changeable moods and their behavioral consequences (3). Mood instability predicts poorer clinical outcomes across mental health disorders such as bipolar disorder (2), including higher rates of relapse into bipolar mood episodes and rates of hospitalization (4,5). Mood instability persists even outside of overt mood episodes and predicts the development of future mood episodes (1). Despite growing recognition of its clinical importance, the mechanisms that underlie the propensity to enter recursive mood cycles in bipolar disorder are poorly understood.

A large body of work has examined the idea of dysregulated reward pursuit in bipolar disorder; specifically, the idea that patients are highly reactive to life events that involve the attainment or failure to obtain reward, potentially resulting in extreme bursts of confidence or excessive goal pursuit (6, 7, 8, 9, 10, 11). Task-based functional magnetic resonance imaging (fMRI) studies have revealed differences in reward sensitivity in bipolar disorder; however, the direction of these effects is highly inconsistent. Even in studies of patients who were not currently experiencing major mood episodes, comparable numbers of studies have found decreased reward sensitivity (12, 13, 14) as have found increased reward sensitivity (15,16). One issue is that studies have typically relied on averaging responses to reward and punishments over entire experiments, whereas the clinical literature suggests a dynamic relationship between mood and reward. Another issue is that reward-based decision making is a multiprocess and recursive phenomenon that comprises anticipatory processes (including expectation), outcome evaluation, and learning and other signals that influence the next decision (17,18). Crucially, while existing studies recognize that these reward processes impact mood, there is emerging evidence demonstrating that changes in mood in turn also impact reward and decision-making processes (19,20).

Computational approaches with a focus on generative models that link unobservable brain states to experimental measurements are well-suited to deriving mechanistic explanations of neuroimaging data (21) and could therefore more precisely capture the dynamics of mood fluctuations. A recent neurocomputational account of bipolar disorder proposed that strong and changeable moods may be underpinned by a propensity for biased perception of reward value, resulting in recursive cycles in which mood, expectations, and behavior oscillate between extremes (22). This explanatory account builds on recent computational fMRI work, which found that violations of expectations in relation to outcomes (i.e., reward prediction errors [RPEs]) drive transient mood fluctuations (19,20).

Eldar and Niv (19) showed that healthy individuals with higher hypomanic traits exhibited increases in striatal activation to reward outcomes when in a positive mood state and decreases when in a negative mood state. Across 2 experiments, they demonstrated support for a generative model consistent with the framework that they have put forward (23). Therefore, this computational account holds great promise for capturing the polar extremes of mood fluctuations such as those seen in bipolar disorder. Under this model, momentary mood is formalized as an integration of recent RPEs, representing an overall momentum of reward in which a higher momentum of rewards and losses engenders a positive and negative mood state, respectively. This quantity was shown to bias the perception of subsequent reward outcomes and better accounted for striatal activation than the standard (unbiased) reinforcement learning model. This model was further corroborated in a subsequent study that probed the mechanisms of action of antidepressant medication (24). In related work, Vinckier *et al.* (20) also demonstrated that momentary mood biases the valuation of potential rewards and losses during decision making. They approximated model-estimated mood fitted from a separate sample and looked for neural correlates in their fMRI data in the absence of mood ratings. Their work also implicated the anterior insula in representing trial-by-trial variation in recent RPE history (mood fluctuations) and reported a negative correlation between model-estimated mood and anterior insular activity. This dovetails with earlier work in which Rutledge *et al.* (25) localized self-reported mood to right anterior insula activation.

Several studies have investigated reward-related striatal functional connectivity in individuals with bipolar disorder and their offspring (i.e., at-risk). Soehner *et al.* (26) found stronger task-based functional connectivity between the ventral striatum and left anterior insula in at-risk individuals than in control participants, which was associated with greater mood dysregulation. In accordance with this, Xi *et al.* (27) showed that resting-state insular-striatal connectivity was positively correlated with the course of illness in individuals with bipolar disorder, highlighting the relevance of insular-striatal connections. However, other studies have also reported decreased striatal connectivity, although with other regions such as the anterior and anteroventral prefrontal cortex in individuals with bipolar disorder (13,28).

Collectively, there is compelling evidence that mood fluctuations can modulate RPE signals represented in the ventral striatum. Recent computational accounts propose that the propensity to enter recursive mood cycles is driven by a stronger mood valuation bias, which may result in the over- or underweighting of value-based options and manifest as dysregulated goal-directed behavior (e.g., high levels of risk-taking or avoidant behavior). These models have great potential for improving understanding of and targeting the mechanisms that underlie the propensity to enter recursive mood cycles, but they have not yet been utilized with clinical populations (22).

The current study puts the central hypothesis that we have outlined previously to the test (22); specifically, individuals with bipolar disorder have an elevated propensity to enter recursive cycles of biased striatal RPEs. We expected that, as in the Eldar and Niv study (19), both groups would show tracking of a signal representing the recent history of outcomes (what we term here as “momentum”) and that this signal would be reflected in the anterior insula (20,25). However, we hypothesized that there would only be a recursive cycle in which momentum biases the perception of subsequent rewards in participants with bipolar disorder. Specifically, we predicted that striatal activations in participants with bipolar disorder, but not matched control participants, would track these momentum-biased RPEs. Finally, we sought to clarify the mechanisms involved by which momentum biases reward perception. We predicted that this propensity would be underpinned by stronger coupling between the ventral striatum and anterior insula in participants with bipolar disorder compared with matched control participants. We predicted that the differences in ventral striatal activation and connectivity would be larger in participants with higher levels of residual mood symptoms.

## Methods and Materials

This study used data from a previously published study (15), which examined how striatal and prefrontal cortical activity was influenced by the probability of reward in bipolar disorder. Importantly, this previous publication did not examine how momentary fluctuations in mood (i.e., momentum) affected reward-related striatal activity using a computational modeling approach, which is the focus of the current study.

### Participants and Power Calculation

Participants were people with bipolar disorder who were not currently experiencing a major mood episode (*n* = 21) and control participants (*n* = 21) matched by age, gender, and level of education (Table 1). The clinical group was recruited from local mental health trusts and specialist affective disorder clinics in Greater Manchester, United Kingdom, and the control group was recruited from the general community, as described in previous work (15). All participants provided written informed consent.

**Table 1:** Participant Demographics and Behavioral Data

	Bipolar Group, *n* = 21	Control Group, *n* = 21	Statistic	*p* Value
Age, Years^a^	35.95 (8.34)	33.25 (9.32)	*t*_38_ = −0.97	.34
Female	11 (52.4%)	9 (42.9%)	χ^2^_1_ = 0.38	.76
Education, Years^a^	14.08 (2.47)	14.70 (2.29)	*t*_38_ = 0.83	.41
Primary Diagnosis				
BD-I	18			
BD-II	3			
Current Comorbidity^a^				
GAD	2			
Lifetime Diagnoses^a^				
AUD/SUD	10			
Panic disorder	4	1		
GAD	2			
OCD	1			
Medications^a^				
Lithium	8			
Valproate	5			
Lamotrigine	2			
SSRI	3			
SNRI	3			
Benzodiazepine	1			
Nonbenzodiazepines	3			
None	4			
HAMD-17	3.83 (2.96)	0.60 (1.04)	*t*_24.90_ = −4.73	<.001
MAS	3.55 (3.09)	0.38 (1.11)	*t*_25.05_ = −4.43	<.001
BIS-11	76.95 (9.94)	62.62 (6.00)	*t*_32.85_ = −5.66	<.001

Values are presented as *n*, *n* (%), mean (SD).

AUD, alcohol use disorder; BD-I, bipolar I disorder; BD-II, bipolar II disorder; BIS-11, Barratt Impulsiveness Scale; GAD, generalized anxiety disorder; HAMD-17, 17-item Hamilton Depression Rating Scale; MAS, Bech-Rafaelsen Mania Scale; OCD, obsessive-compulsive disorder; SNRI, serotonin-norepinephrine reuptake inhibitor; SSRI, selective serotonin reuptake inhibitor; SUD, substance use disorder.

a Data from 2 participants are missing and not included.

The main inclusion criteria were 18 to 45 years of age, weekly alcohol intake below 26 units, and no substance use for 4 months before the study. The Structured Clinical Interview for DSM-IV Axis I disorders (29) was used to confirm the diagnosis of bipolar disorder and screen control participants. Individuals who did not meet the threshold for either manic or depressive episodes (i.e., out-of-episode) for 2 months before the study were included.

Individuals were excluded if they had received antipsychotic medication 6 months before the study. Clinicians assessed residual manic and depressive symptoms using the 11-item Bech-Rafaelsen Mania Scale (MAS) (30) and the 17-item Hamilton Depression Rating Scale (31), respectively. The authors of the MAS have reported good internal consistency (α = 0.80–0.90), and the 17-item Hamilton Depression Rating Scale has similarly been shown to have good internal consistency in a sample of individuals with unipolar and bipolar depression (α = 0.71–0.85) (32,33). Trait impulsivity was assessed using the 30-item Barratt Impulsiveness Scale (BIS-11) (34), which has also been reported to have good internal consistency (α = 0.83) (35).

Power analysis was informed by Eldar and Niv’s study (19) and is described in detail in the Supplement. We conducted a power calculation using G∗power (36) that yielded an estimate of 89% implied power with our sample of 42 participants at an alpha level of 0.05.

### Task

**Figure 1:**
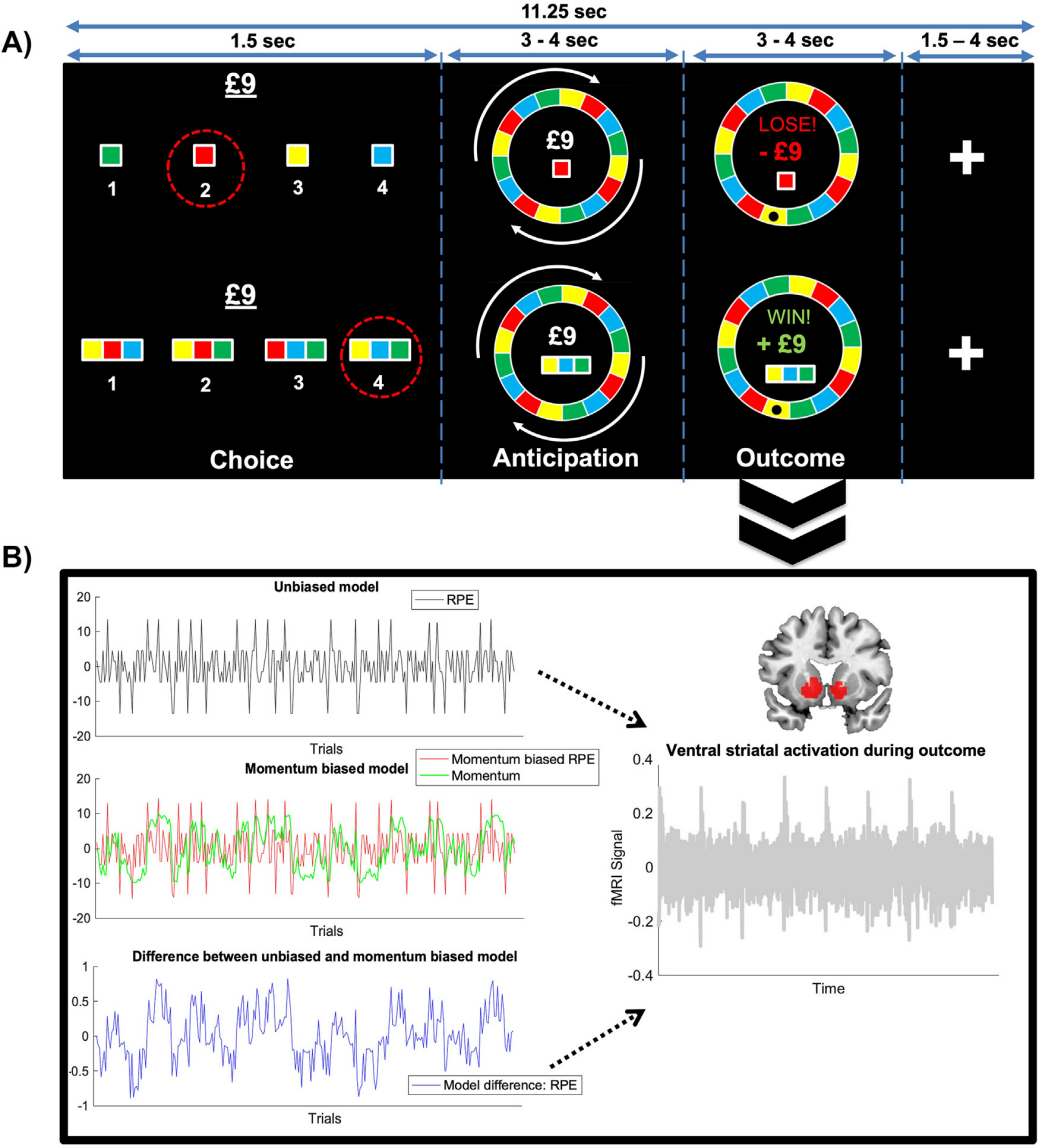
Schematic of the Roulette task and model-based functional magnetic resonance imaging. **(A)** Roulette task. Participants placed bets on which color would win in a Roulette spin in each trial, and the probabilities (25% or 75%), stakes (£3 or £9), and outcomes were pseudorandomized within blocks of 34 trials such that these conditions were equally balanced across all participants. The red dotted circles during choice correspond to an example choice a participant could make under a low (25%) or high (75%) probability of reward. After betting, the wheel began spinning (anticipation) before stopping, and then participants received feedback (outcome). **(B)** Model-based functional magnetic resonance imaging with parametric modulators for trialwise reward prediction error (RPE) during outcome evaluation. The RPE time series was generated under a standard, unbiased model and under a recursive biased model in which the momentum of recent outcomes impacted the perception of subsequent outcomes. Adapted with permission from Mason *et al.* (15).

Participants completed a variant of a validated Roulette task (37) in which reward probability and magnitude were independently manipulated. Each trial consisted of 3 stages: choice, anticipation, and outcome (Figure 1). During choice, participants chose between 4 options that conferred the same probability of reward. In low-probability trials (25% chance of reward), participants selected 1 of 4 individual colors that made up the Roulette wheel. In high-probability trials (75% chance of reward), they selected between 4 sets of 3 colors and won if the Roulette wheel stopped on any one of the 3 colors. The stake on offer (reward magnitude) was presented prior to choice, with equal numbers of low- (£3) and high- (£9) magnitude trials. During anticipation, the Roulette wheel spun (3–4 seconds). At outcome, the wheel stopped spinning, with the location of the Roulette ball indicating whether the participant had won or lost the amount of money at stake.

Participants were instructed to respond within the fixed duration of the choice phase; otherwise, a random choice would automatically be selected for that trial. Participants were informed that they would be paid the actual winnings following task completion. Participants completed 272 trials across 8 blocks, each lasting approximately 6 minutes.

### fMRI Acquisition and Preprocessing

Standard fMRI acquisition was conducted as reported in previous work (15) and is described in the Supplement. The ArtRepair toolbox (38) was used to minimize the impact of artifacts via the interpolation of outlier volumes, which resulted in the inclusion of fMRI data from all 42 participants.

### Model-Based fMRI

First-level analyses were a general linear model implemented in SPM12. fMRI blood oxygen level–dependent responses for each participant were modeled using regressors representing the 3 task conditions (choice, anticipation, and outcome), 2 parametric modulators modeled at outcome (trialwise unbiased RPEs and momentum-biased RPEs), and 6 motion realignment parameters to reduce residual motion effects, yielding 11 regressors.

**Figure 2:**
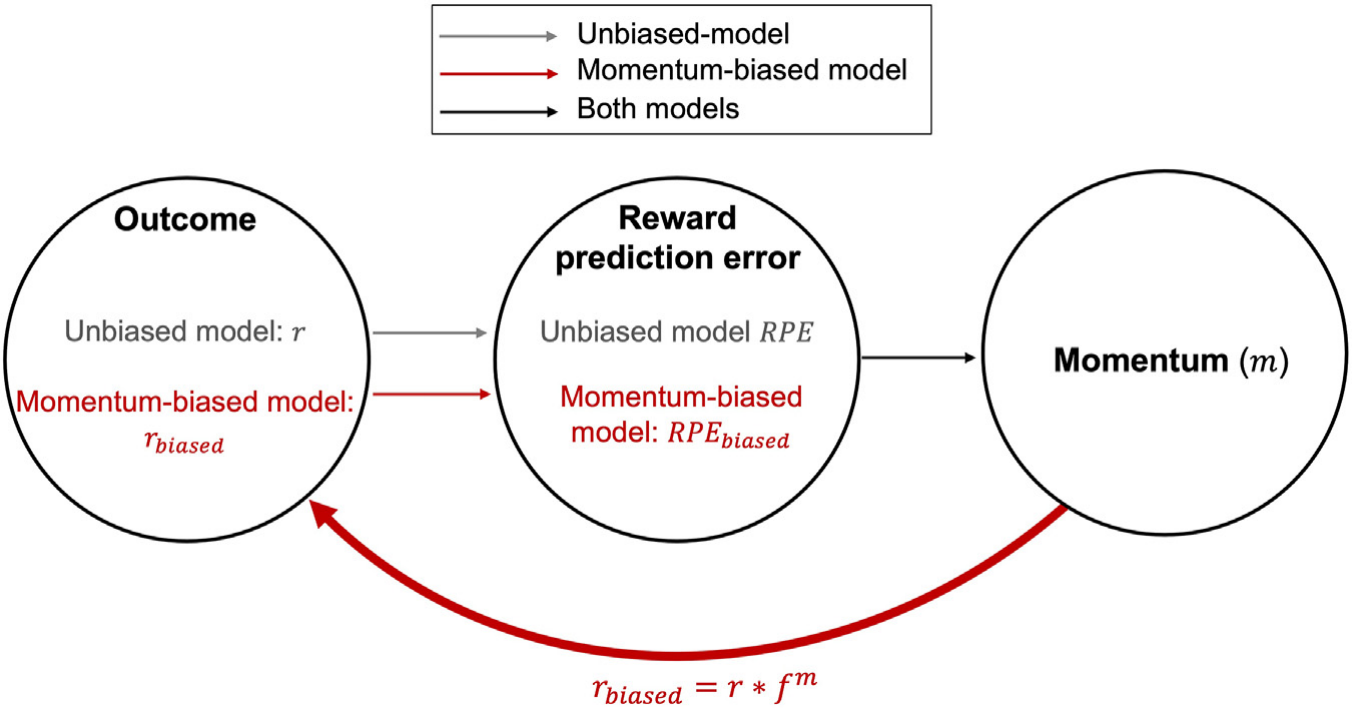
Illustration of unbiased and momentum-biased models. RPE, reward prediction error.

We generated trialwise unbiased and momentum-biased RPE values from Eldar and Niv’s computational model (19), where momentary mood is formalized as the accumulation of recent outcomes (i.e., momentum of RPEs) and influences the trialwise RPE. Specifically, we computed unbiased RPEs from the expected values and outcomes received by participants. Then, we generated momentum-biased RPEs under a generative model in which recent RPEs are allowed to summate and bias the perception of subsequent outcomes (Figure 2). Given evidence of impaired reinforcement learning in bipolar disorder (39,40), we utilized a task that removed learning, which precluded fitting exact individual participant parameters. Instead, we sought to quantify the extent to which ventral striatal activations were consistent with a momentum-biased agent, utilizing the group average momentum bias parameter in participants with elevated hypomanic personality traits, previously reported by Eldar and Niv (19) (see the Supplement). This approach is consistent with recommendations for model-based fMRI (41), which showed that effects when regressing RPE time series generated under a single average parameter for all participants are comparable to the time series generated under individual parameter estimates (see the Supplement).

Following Eldar and Niv’s approach (19), momentum-biased RPE was calculated as the difference between unbiased and momentum-biased estimates to avoid regressor collinearity. Regressors were convolved with a canonical hemodynamic response function. Individual contrast images of interest (per-trial estimates of unbiased and momentum-biased RPE) were computed and passed to a second-level general linear model to examine within-group and between-group activations. We also included model-estimated values of momentum as a parametric modulator in a separate general linear model to corroborate that this signal was represented in the anterior insula, consistent with previous studies that have localized momentary mood to anterior insula activations (20,25).

As a confirmatory check, we conducted 2 analyses, which tested 1) a model without computational modeling assumptions to confirm a carryover effect of RPEs from the previous trial (RPE_t − 1_) to the next trial (RPE_t_), and 2) a simpler model, where RPE history is allowed to accumulate farther back than RPE_t − 1_ with only a momentum-update parameter and no momentum-bias parameter. The findings indicated that both RPE history and the interaction term were only tracked in participants with bipolar disorder and not in matched control participants (see the Supplement).

### Striatal Tracking of Momentum-Biased RPEs

Analyses utilized a region of interest (ROI) approach. The procedure by which we defined our ventral striatum and left and right anterior insula ROIs is described in the Supplement. Parameter estimates representing the mean activity across all voxels in these ROIs for each participant were extracted for each contrast of interest using MarsBaR (http://marsbar.sourceforge.net/) using a standard threshold (*p* < .05). For the ventral striatum ROI, the contrast of interest was the parametric modulation of outcome-locked activation by trial-by-trial momentum-biased RPE values (i.e., the difference between unbiased and momentum-biased estimates). For the left and right anterior insula ROIs, the contrast of interest was the parametric modulation by trial-by-trial momentum, and Bonferroni correction was used with these 2 tests.

To test for associations with residual mood symptoms, manic and depressive symptoms were entered as predictors into a regression analysis in participants with bipolar disorder. Trait impulsivity was accounted for in this analysis because it had previously been shown to impact reward activations in this dataset (15). Log transformation was used for non-normal variables.

### Functional Connectivity of Striatal Momentum-Biased RPE Activations

To examine insular-striatal connectivity as a locus of momentum biasing reward perception, we quantified the extent to which outcome-locked functional connectivity between these 2 regions was modulated by momentum-biased RPEs. We used generalized psychophysiological interaction analysis (http://www.nitrc.org/projects/gppi) (42) with our ventral striatum ROI as the seed. At first level, the fMRI time series for outcome-locked activations was extracted from the seed region for each run and multiplied by the task regressor of interest to form the psychophysiological interaction terms. Second-level analyses were conducted to identify within- and between-group connectivity between the ventral striatum and anterior insula using MarsBaR. The contrast of interest for the functional connectivity analyses was the parametric modulation of outcome-locked activation by trial-by-trial momentum-biased RPE values (i.e., the difference between unbiased and momentum-biased estimates).

## Results

Participant demographic characteristics and behavioral results are reported in Table 1. Compared with matched control participants, participants with bipolar disorder showed higher levels of manic (MAS) and depressive (17-item Hamilton Depression Rating Scale) symptoms and trait impulsivity (BIS-11). Objective momentum of reward statistics did not differ between the groups (*p* ≥ .31) (Supplement; Figure S1).

### Striatal Tracking of Momentum-Biased RPEs

Ventral striatal activation during anticipation and outcome has been reported in detail elsewhere (15); both were greater in participants with bipolar disorder than in matched control participants (see the Supplement).

Consistent with previous literature, bilateral ventral striatal activation tracked unbiased RPE across participants with bipolar disorder (mean = 0.28, SD = 0.22, *t*_20_ = 6.16, *p* < .001, *d* = 1.27) and matched control participants (mean = 0.20, SD = 0.14, *t*_20_ = 6.04, *p* < .001, *d* = 1.42).

**Figure 3:**
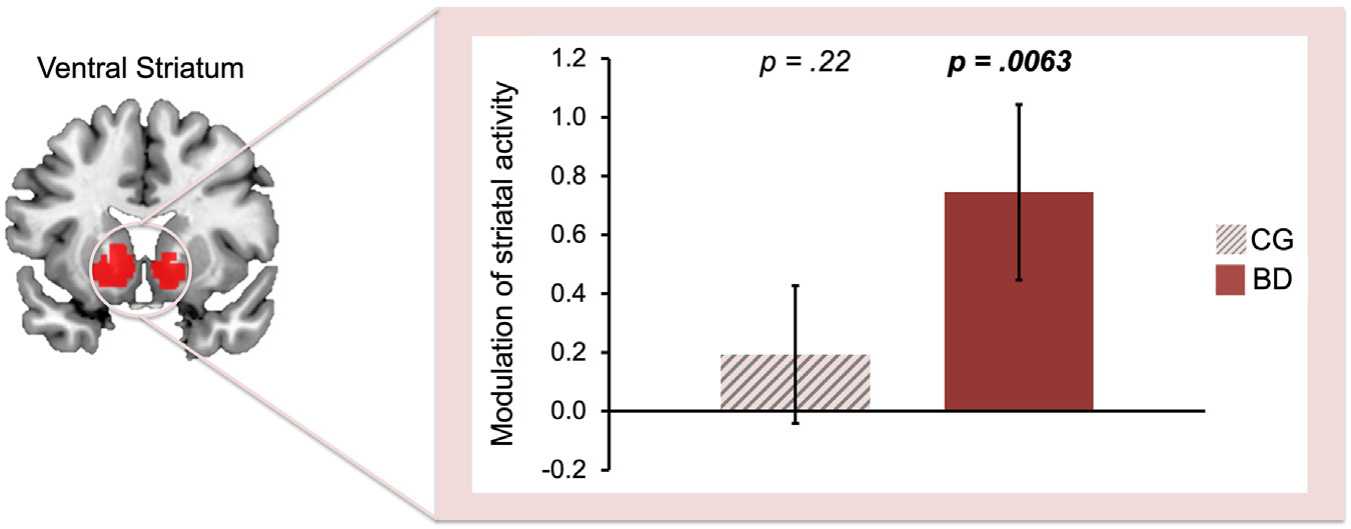
Modulation of outcome activity by momentum-biased reward prediction errors in the ventral striatum. Only the participants with bipolar disorder tracked momentum-biased reward prediction errors (error bars: standard error of the mean). BD, bipolar disorder group; CG, control group.

Bilateral ventral striatal activation tracked momentum-biased RPE only in participants with bipolar disorder (mean = 0.75, SD = 1.37, *t*_20_ = 2.61, *p* = .0063, *d* = 0.54) and not in matched control participants (mean = 0.19, SD = 1.08, *t*_20_ = 0.78, *p* = .22, *d* = 0.18) (Figure 3), which partially corroborated our hypothesis. Participants with bipolar disorder also trended toward showing greater tracking of momentum-biased RPE than matched control participants, but this did not reach significance (*t*_40_ = 1.46, *p* = .075, *d* = 0.46). Hypomanic personality traits are normally distributed in the general population (43), with bipolar disorder lying at the extreme of this continuum. Eldar and Niv (19) performed a median split on a sample drawn from the general population and found significant differences in biased reward perception. We reasoned that we would see comparable differences in biased reward perception in our control group, potentially hampering group differences when comparing the control group with the bipolar disorder group. In fact, we found evidence for this (see the Supplement and Figure S2).

This momentum-biased RPE-modulated striatal activity was not predicted by levels of manic and depressive symptoms in participants with bipolar disorder (Supplement; Table S1).

### Insular Tracking of Momentum

**Figure 4:**
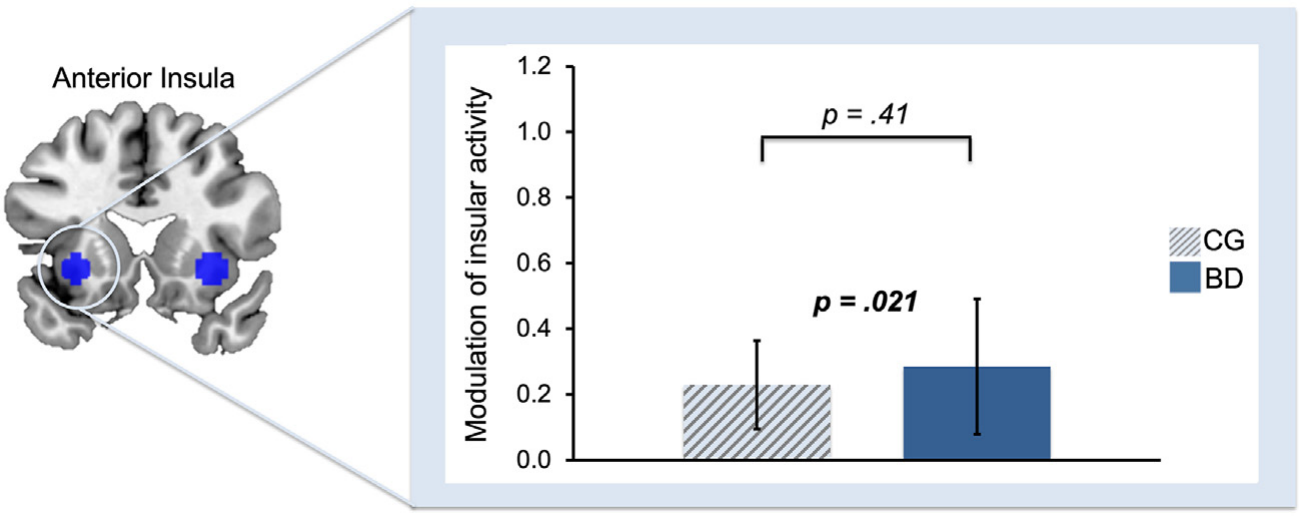
Activation in the left anterior insula tracked the degree of momentum of reward prediction errors. In both groups, momentum was tracked by the left anterior insula (*N* = 42, *p* = .021), and this was not significantly different between groups (*p* = .41) (error bars: standard error of the mean). BD, bipolar disorder group; CG, control group.

In both groups, we confirmed that momentum (i.e., the aggregate of recent outcomes) (see Methods and Materials) was tracked in the anterior insula (left anterior insula selectively, *t*_41_ = 2.11, *p* = .021, *d* = 0.33 and not the right anterior insula, *p* = .31, *d* = 0.08; Bonferroni-corrected *p*-value threshold is .025) (Figure 4). As expected, we did not find a group difference in the degree to which the anterior insula tracked momentum (*p* ≥ .41). Results for insular tracking of unbiased RPEs are reported in the Supplement.

### Modulation of Striatal Connectivity by Momentum-Biased RPEs

Next, we sought to understand why ventral striatal activations tracked momentum-biased RPEs only in participants with bipolar disorder (Figure 3) and whether this was accompanied by differences in the integration of the momentum signals that we identified in left anterior insula activation. To do this, we quantified the degree to which the coupling between the ventral striatum and anterior insula was modulated by fluctuations in momentum. We focused on the left anterior insula because there was left-lateralized selectivity in tracking momentum.

**Figure 5:**
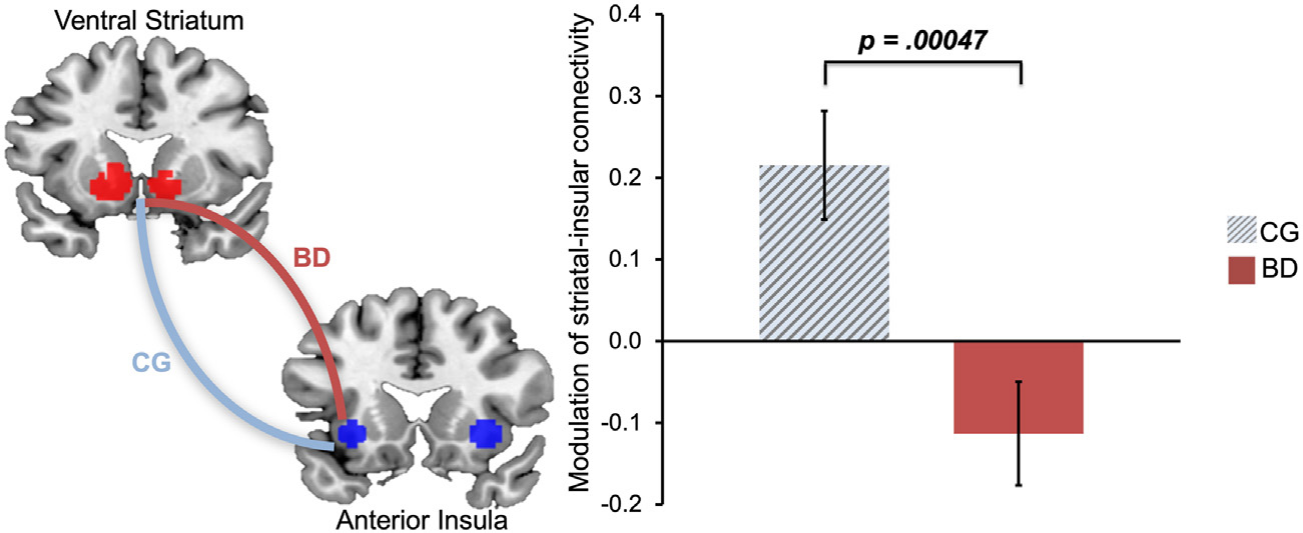
Divergent modulation of left anterior insular-ventral striatal functional connectivity by momentum-biased reward prediction errors in participants with bipolar disorder compared to matched controls. Functional connectivity between the left anterior insula and bilateral ventral striatum during outcome (error bars: standard error of the mean). The blue line represents modulation in the CG, and the red line represents modulation in the BD. BD, bipolar disorder group; CG, control group.

We found that participants with bipolar disorder diverged from matched control participants in how their ventral striatal connectivity with the left anterior insula was modulated by momentum-biased RPEs (*t*_40_ = 3.38, *p* = .00047, *d* = 1.07). However, this group difference was in the opposite direction from our hypothesis. Whereas control participants showed a significantly stronger positive modulation of ventral striatal connectivity with the left anterior insula (mean = 0.22, SD = 0.31, *t*_20_ = 3.24, *p* = .0012, *d* = 0.71), participants with bipolar disorder showed modulation in the opposite direction (mean = −0.11, SD = 0.30, *t*_20_ = −1.78, *p* = .041, *d* = −0.37). Moreover, the divergent negative coupling was greater in individuals with elevated residual manic symptoms (Figure 5) and as a function of trait impulsivity (MAS: β = −0.45, *t*_17_ = −2.08, *p* = .05; BIS-11: β = 0.47, *t*_17_ = 2.26, *p* = .04) (Supplement; Table S2).

For specificity, we confirmed that left insular-striatal connectivity was not modulated by unbiased RPE in either group (*p* ≥ .13).

## Discussion

This study put the theory that bipolar disorder is underpinned by a propensity for reward perception to be biased by fluctuations in momentum of recent RPEs to the test.

Consistent with the hypothesis put forward in our previous work (22), we found evidence that ventral striatal RPEs were excessively modulated by momentum in individuals with bipolar disorder (Figure 3). Moreover, they showed divergent modulation of left anterior insular-ventral striatal connectivity, the extent of which increased as a function of current manic symptoms.

Consistent with previous work, we found that both participants with bipolar disorder and matched control participants tracked the momentum of recent RPEs (Figure 4). This quantity, previously shown to capture momentary mood (19), was tracked by the anterior insula, a region implicated in 2 other studies that examined the intersection of mood and reward processes (20,25). However, only participants with bipolar disorder showed evidence that this momentum quantity additionally biased RPE signals in the ventral striatum (Figure 3). Thus, we found evidence for the hypothesis that a bidirectional relationship between mood and reward processes exists in bipolar disorder, such that fluctuations in momentum bias the perception of subsequent outcomes in a recursive cycle. We have previously argued, through simulations, that this mechanism is sufficient to drive the oscillatory changes in mood that culminate in manic and depressive episodes (22).

We sought to deepen this insight by asking how momentum might impact striatal RPEs in bipolar disorder. We reasoned that this would come about through excessive coupling between the anterior insula and ventral striatum, consistent with an overpropagation of the momentum signal. We found that insular-striatal functional connectivity diverged in bipolar disorder, but this was due to stronger negative coupling between these regions as a function of momentum-biased RPEs. Thus, our findings suggest that in periods of upward momentum (better-than-expected outcomes), insular-striatal coupling became stronger in matched control participants and weaker in participants with bipolar disorder.

**Figure 6:**
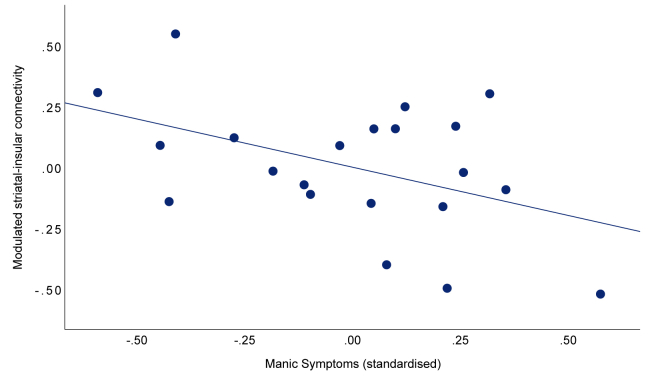
Stronger inverse modulation of left anterior insular-ventral striatal connectivity as a function of residual manic symptoms in participants with bipolar disorder. Partial association from regression model of functional connectivity between the left anterior insula and bilateral ventral striatum modulated by momentum-biased reward prediction errors. Log-transformed scores from the Bech-Rafaelsen Mania Scale.

While unexpected, one interpretation of the connectivity results is that greater coupling with the anterior insula serves to contextualize (rather than bias) reward perception. By holding a running total of momentum alongside new outcomes, this could suppress signals that would otherwise provide recursively confirmatory evidence that the environment is changing. In participants with bipolar disorder, the failure to contextualize, through strengthened positive connectivity during periods of upward momentum (better-than-expected outcomes), could increase susceptibility to misperceive upward momentum and to act on it, further escalating mood. Consistent with this possibility, we found this divergent connectivity to be even stronger in participants with bipolar disorder with higher levels of manic symptoms (Figure 6), consistent with overconfidence and risk taking during (hypo)mania (44,45). It is possible that the unexpected findings from the generalized psychophysiological interaction analyses are due to large group differences that existed during the anticipation and outcome phases [c.f. (12, 13, 14,46,47)], which may have resulted in less residual variation in the time series that is picked up by unbiased and momentum-biased RPEs. Therefore, the interpretation that the divergent insular-striatal connectivity observed in participants with bipolar disorder is linked to reduced contextualization of upward momentum remains speculative until corroborated by additional work; however, we confirmed that the insular-striatal coupling was specifically modulated by momentum-biased RPEs and not unbiased RPEs.

Intriguingly, we observed that higher levels of trait impulsivity (BIS-11) offset the inverse striatal-insular coupling that we observed in participants with bipolar disorder. According to the above interpretation, trait impulsivity offsets the tendency to integrate the momentum of past rewards when evaluating current outcomes. Impulsivity has long been conceptualized as a myopic focus on immediate rewards or a failure to integrate past temporal horizon (48,49) including disproportionate striatal activations for decisions that involve immediate rewards (50). Previous analyses using the current dataset revealed that impulsivity predicted reduced reactivity to safe (high-probability) rewards beneficial for longer-term goals (15). Other measures that tap emotion-related impulsivity (e.g., engaging in rash behavior following positive or negative emotions) have been highlighted as being more relevant to frequent and intense mood oscillations observed in bipolar disorder (51,52). Consistent with this notion, we argue that we could be picking up a distinct dimension of impulsivity (beyond inattentive or nonplanning impulsivity) in bipolar disorder, which may be an epiphenomenon of being excessively prone to momentum.

A limitation of the current study is that while we assessed current mood symptoms on the day of the scan, we did not obtain ratings of momentary mood during the task. This would be useful to confirm that momentum corresponds to momentary mood, as found previously in participants with elevated hypomanic personality traits (19) and in general population samples (20). Consistent with previous reports that frequent and intense fluctuations in mood persevere outside of mood episodes (1), our sample had residual mood symptoms, which limits the conclusions that can be drawn about the extent to which the group differences are due to trait or state. Importantly, the core finding of excessive striatal tracking of momentum-biased RPEs was independent of current mood symptoms, indicating that this may be a core feature of bipolar disorder. In contrast, only the functional connectivity findings were modulated by clinical state, perhaps indicating that the putative regulation mechanism we outlined above is further impaired by clinical state. Additional research is needed to assess whether divergent functional connectivity is involved in the escalation of manic symptoms. Finally, participants in our sample were receiving psychiatric medication. While previous reviews have concluded that medications pose minimal confounds for neuroimaging studies (53,54), this could nonetheless have reduced our power to detect group differences. On the other hand, we did not include, by design, participants who were receiving antidopaminergic medications. Given the dopaminergic innervation of the loci we identified in the current study, it will be intriguing to evaluate whether the efficacy of this class of medication (55,56) occurs via amelioration of momentum-biased RPE signaling.

### Conclusions

We identified excessive striatal tracking of momentum-biased RPEs as one of the potential core mechanisms in bipolar disorder, consistent with theoretical accounts (22). We also found divergent striatal-insular coupling during periods of upward momentum, as a putative marker for poorer contextualization of reward perception, and exacerbation of manic symptoms. This sets the stage for evaluating this mechanism as a novel target that may be amenable to intervention in bipolar disorder.

## Supplementary Material

Key Resources Table
