# Supplementary material for: Misperceiving Momentum: Computational Mechanisms of Biased Striatal Reward Prediction Errors in Bipolar Disorder
Source: Biol Psychiatry Glob Open Sci. 2024 Apr 30;4(4):100330. doi: 10.1016/j.bpsgos.2024.100330 (PMC11313182; doi:10.1016/j.bpsgos.2024.100330)
Supplement: Supplement [file mmc2.pdf]

SUPPLEMENTARY INFORMATION

Misperceiving Momentum: Computational Mechanisms of Biased Striatal Reward Prediction Errors in Bipolar Disorder

Moningka and Mason

This file includes:

1. Supplementary Methods
2. Supplementary Tables and Figures
3. Supplementary Results
4. Supplementary Discussion

1. Supplementary Methods

Power calculation

Power analysis was informed by Eldar and Niv's study, which compared striatal and ventromedial prefrontal cortex activity between healthy participants exhibiting low versus high hypomanic traits, as defined by a median split on the Hypomanic Personality Scale. Using a similar reward-based task, they found a significant difference in striatal activations between participants with high and low hypomanic traits with an effect size of d = .90, which we used to determine the implied power in our study. We conducted a power calculation using G*power and yielded 89% implied power with our sample of 42 participants at an alpha level of .05. Since our study examines striatal activation between individuals with bipolar disorder and matched controls, the effect size obtained should be significantly larger than those obtained in Eldar and Niv's non-clinical sample.

FMRI acquisition and preprocessing

Echo-planar image sequence with repetition time = 2450ms, echo time = 25ms, flip angle = 90°, slices = 30 in ascending order, slice thickness = 4mm, in-plane resolution = 1.5 x 1.5mm and a standard field-of-view was acquired from a 1.5T Phillips scanner. Data were processed using SPM12 (Wellcome Centre for Human Neuroimaging, University College London) and Matlab R2019b. Each participant's functional images were motion-corrected using a six-parameter rigid-body transformation to the mean image and slice-time-corrected to the middle slice. Functional images were co-registered with each participant's structural image, spatially normalised to the Montreal Neurological Institute (MNI) standard template and smoothed using an 8mm Gaussian

kernel. Intrinsic autocorrelations were accounted for by AR(1) and low frequency drifts were removed via the 128s high pass filter.

ROI definition

To define the ventral striatum ROI, we followed Eldar and Niv's approach and included all grey-matter voxels within the bilateral ventral striatum that responded to 'reward' outcomes versus 'loss' outcomes contrast at a family-wise-error (FWE) corrected threshold of *p*FWE < .05 (1). We extracted activations from an insula ROI, defined as 8mm spheres around the peak coordinates for the bilateral anterior insula reported by Vinckier and colleagues (4) (left: x = -30, y = 22, z = -6; right: x = 32, y = 20, z = -6).

Supplementary formula

Unbiased reward perception model

In the unbiased model, reward expectations were formalised as the net expected value (EV) of the possible outcomes; that is the probability and value of winning the sum of money at stake combined with the probability and value of losing this stake. Reward prediction error (RPE), was operationalised as the difference between the actual outcome value (r) obtained and the expected value i.e. $RPE = r - EV$. Multiple studies have shown that RPEs are still tracked in tasks without learning components (5, 6). EV estimates on the next trial are not updated because EV estimates are explicit on every trial and RPE does not drive the update to EV.

Momentum-biased reward perception model

To account for effects of momentum on each trial ($m_{(t)}$) on valuation, the unbiased model is modified to compute momentum-biased RPEs using perceived momentum-biased outcome value (r_{biased}) instead of the objective outcome (r) (1).

$$r_{biased} = r \,.\, f^{m} \tag{1}$$

$$RPE_{biased} = r_{biased} - EV \tag{2}$$

Here, f is the momentum bias parameter that indicates the direction and degree of momentum bias. If f = 1, momentum does not bias the perception of reward or expected value. With f > 1, momentum exerts positive feedback i.e. reward is perceived as larger in a good mood and smaller in a bad mood, whereas 0< f <1 would correspond to a negative feedback on reward value. In our present study, we set f to 1.2, based on the average f derived from Eldar and Niv's (1) sample of control participants who scored relatively high on the Hypomanic Personality Scale (2), a measure of trait mood instability, as determined by a median split.

As per Eldar and Niv's model (1), we updated momentum on each trial, ($m_{(t)}$), based on the momentum at the beginning of the trial, ($m_{(t-1)}$), and the difference between RPE on the current trial, $RPE_{(t)}$ and $m_{(t-1)}$ (i.e. the degree to which the environment is improving or worsening).

$$m_{(t)} = m_{(t-1)} + \eta(RPE_{(t)} - m_{(t-1)}) \tag{3}$$

Before applying momentum to r, we constrained it using a sigmoid function, allowing it to take values between -1 and 1:

$$m = \tanh(m) \tag{4}$$

Unlike Eldar and Niv's study (1), we did not use subjective ratings of mood to as a confirmatory check of how well the model captures participants' self-reported mood during the task. However, Eldar and Niv's (1) findings lend support to this model; it outperformed the unbiased and other reinforcement-learning models and explained participants' trial-by-trial choices and subjective mood ratings well. We also conducted additional analyses (see Supplementary Results) to test two models with fewer assumptions. Specifically, we tested a GLM with no assumptions about computational model parameters, in which RPE in the previous trial (i.e. RPE_{t-1}) predicts outcome-locked striatal and insular activation in the next trial, on top of RPE_t. We also tested a computational model in which we removed momentum bias and instead examined the influence of the interaction of RPE history with RPE, analogous to biased RPE_t in our main analyses. We found that in both these cases only participants with bipolar disorder track 1) momentum (i.e. RPE_{t-1}) and 2) biased RPE derived from the simplified model without a momentum bias parameter.

Given that in our task, the probability and magnitude of outcomes were fixed and made explicit in each trial, the RPEs do not have utility for updating expectations. Instead, the model updates trial-by-trial estimates of momentum (m) using averaged free parameter values from previous datasets using this model (1). Given this, we did not directly quantify momentum and momentum-update rate, which quantifies how quickly

momentum is updated from trial-to-trial, from our task. We imposed group-level values on all participants from a previous study using participants with high hypomanic traits (1), which may not fully capture inter-individual variability. We reasoned that the average parameter values would be similar in our bipolar group to the high hypomanic participant group given that high hypomanic symptoms confer psychometric vulnerability for bipolar disorder (2). However, Wilson and Niv (7) demonstrate that deriving RPEs under group-averaged learning rate parameters achieved comparable fits to the fMRI data as utilising individually estimated learning rate parameters. Moreover, they found that, for learning rate, the quality of fit of the resulting RPEs to ventral striatal activations was highly robust even to large deviations from the actual group average. However, these specific results pertain to the effect of learning rate and given our present findings, future research could examine this in relation to the momentum bias parameter.

2. Supplementary Tables and Figures

Table S1. Linear regression coefficients with manic and depressive symptoms and impulsive traits as predictors of ventral striatal activity modulated by momentum-biased RPE in participants with bipolar disorder.

Predictor	*B*	95% CI for *B*	β	*t*	*p*	Tolerance	VIF
Intercept	.44	[-.44, 1.32]		1.05	.31		
MAS	-.08	[-.45, .29]	-.12	-.46	.65	.84	1.19
HAMD-17	-.007	[-.05, .03]	-.09	-.36	.72	.91	1.10
BIS-11	.001	[-.01, .01]	-.05	-.21	.84	.91	1.11

R^2 = .04, n = 21, $F(3,20)$ = .22, p = .88. MAS = Bech-Rafaelsen Mania Scale; HAMD-17 = Hamilton Depression Rating Scale; BIS-11 = Barratt Impulsiveness Scale; CI = confidence interval; VIF = Variance Inflation Factor.

Table S2. Linear regression coefficients with manic and depressive symptoms and impulsive traits as predictors of left anterior insular-ventral striatal functional connectivity modulated by momentum-biased RPE in participants with bipolar disorder.

Predictor	*B*	95% CI for *B*	β	*t*	*p*	Tolerance	VIF
Intercept	-.92	[-1.90, .04]		-2.02	.06		
MAS	-.40	[-.80, .01]	-.45	-2.08	.05	.84	1.19
HAMD-17	-.01	[-.05, .03]	-.11	-.51	.62	.91	1.10
BIS-11	.01	[.00, .03]	.47	2.26	.04	.91	1.11

R^2 = .32, n = 21, $F(3,20)$ = 2.72, p = .08. MAS = Bech-Rafaelsen Mania Scale; HAMD-17 = Hamilton Depression Rating Scale; BIS-11 = Barratt Impulsiveness Scale; CI = confidence interval; VIF = Variance Inflation Factor.

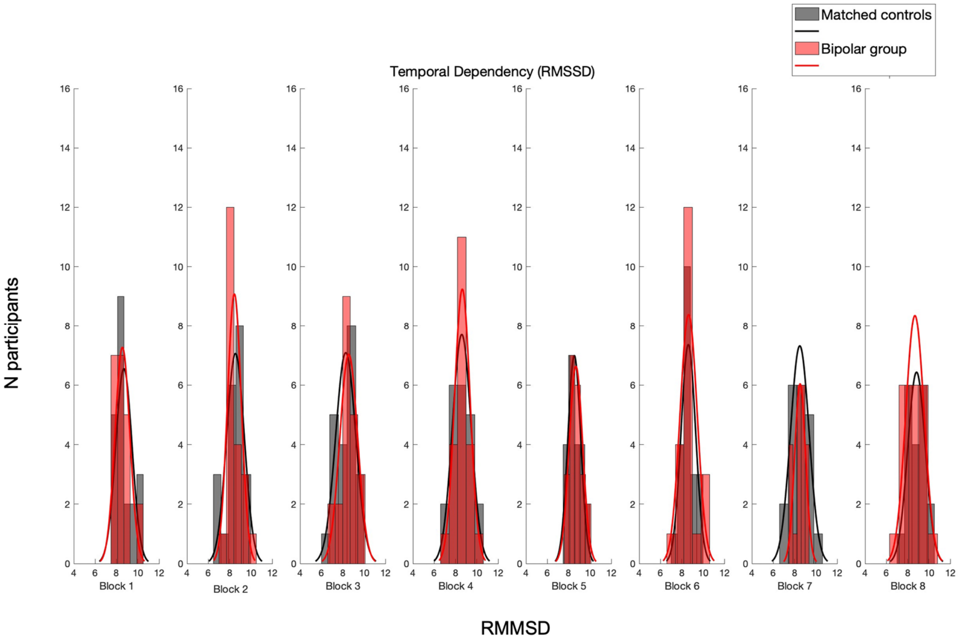

Figure S1. Histogram depicting temporal dependency as measured by root mean square of successive differences (RMSSD) between objective reward statistics (RPE). This is shown across 8 blocks of 34 trials in matched controls (green bars) and participants with bipolar disorder (red bars). There were no statistically significant differences in temporal dependency between groups (Block 1: t = .59, p = .56; Block 2: t = .44, p = .67; Block 3: t = -1.03, p = .31; Block 4: t = -.21, p = .84; Block 5: t = -.81, p = .42; Block 6: t = -.13, p = .90; Block 7: t = -.15, p = .88; Block 8: t = .56, p = .58).

3. Supplementary Results

Confirmatory checks

We conducted confirmatory checks which relied on fewer modelling assumptions to determine whether there is a carry-over effect of "momentum" in participants with bipolar disorder. Specifically, we tested 1) whether the RPE value in the previous trial (i.e. RPE_{t-1}) predicts outcome-locked striatal activation on the current trial, t (after confirming that these values were uncorrelated: median r = -.064), and 2) whether this effect is stronger in participants with bipolar disorder. This is analogous to the momentum-bias of RPE in the computational modelling analyses. This analysis confirmed that only participants with bipolar disorder tracked RPE_{t-1} in the ventral

striatum [Control group: $t(20) = 1.32$, $p = .10$, $d = .29$; Bipolar group: $t(20) = 2.62$, $p = .006$, $d = .57$]. Thus, RPE_{t-1} ("momentum") predicts ventral striatal activity on top of RPE_t, suggesting a carry-over effect of RPE from the current trial to the next trial in participants with bipolar disorder. Moreover, RPE_{t-1} is represented in the left anterior insula only in the bipolar group [$t(20) = 2.11$, $p = .02$, $d = .46$] which might suggest excessive tracking of momentum in brain areas involved in affective processing.

We also tested a simplified computational model in which we removed the momentum bias parameter, to see whether striatal activation was additionally modulated by the interaction between RPE history and current RPE. This analysis also converged in finding evidence that this interaction term was only tracked in the bipolar group [$t(20) = 2.51$, $p = .008$, $d = .52$] and not in matched controls [$t(20) = .57$, $p = .29$, d = .18].

Striatal activity during anticipation and outcome (results from Mason et al., 2014)

For the anticipation stage, a three-way group by probability hemisphere interaction approached significance ($p = 0.058$, $d = .63$) and showed that left ventral striatal activity was greater in participants with bipolar disorder than matched controls. For the outcome stage, an overall group effect was found ($p = 0.04$, $d = .69$); follow-up analyses showed greater activity for gains (vs. losses) ($p < 0.001$, $d = .75$) and large (vs. small) outcomes ($p = 0.024$, $d = .76$).

Striatal tracking of momentum-biased RPE (within matched controls)

Our matched control group is comparable to the entire non-clinical sample in Eldar & Niv (2015), i.e. their low and high bias subgroups combined. Our effect size within our

bipolar group is indeed larger (d = 0.54) than that in the high-bias group reported by Eldar and Niv (2015; t-value of only 0.69±0.15, Cohen's d = 0.29 assuming df = n-1 = 11.5 on median split of total n = 25). This is to be expected given that ours is a clinical sample. However, the effect of biased reward perception in our matched control group is many times larger (d = .18) than the effect in their low-bias group (d = 0.02, based on "t = −0.04±0.14", df = 11.5. Indeed, when we split our matched controls by score on the Hypomanic Personality Scale (see figure below), to derive something comparable to the low and high subgroups in Eldar and Niv (2015), the effect sizes in our lower HPS subgroup are much weaker (d= 0.06) than in our higher HPS subgroup (d = 0.30). Consequently, our matched controls have an effect size comparable to the net of the two subgroups in Eldar and Niv (2015), thereby potentially hampering a between-group difference for our bipolar participants compared to our control group as a whole.

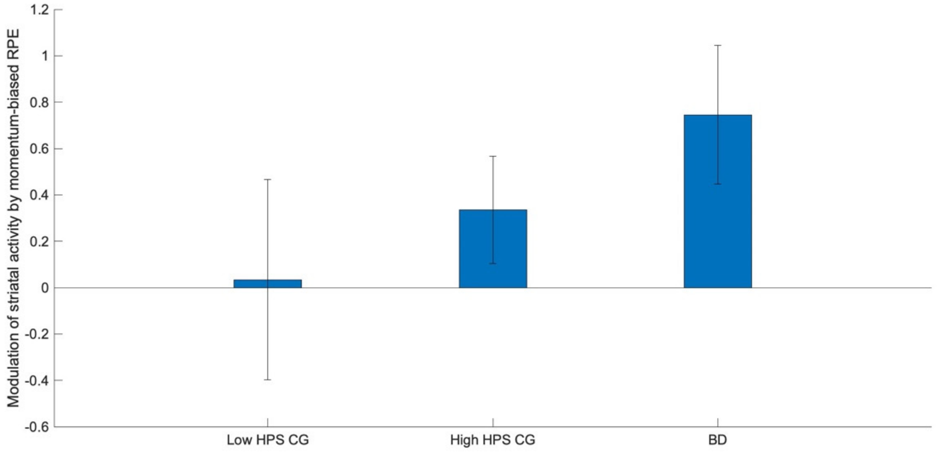

Figure S2. Mean striatal activity modulated by momentum-biased RPE across two subgroups of matched controls that had low (n=10) and high scores (n=11) on the Hypomanic Personality Scale (as determined by a median split) and participants with bipolar disorder (n=21). CG = Control Group; BD = participants with bipolar disorder.

Insular tracking of unbiased RPE

Left anterior insula activation tracked unbiased RPE across both participants with bipolar disorder [$M = .20$, $SD = .22$, $t(20) = 4.15$, $p = .000083$, $d = .91$] and matched controls [$M = .11$, $SD = .17$, $t(20) = 2.83$, $p = .0036$, $d = .72$]. Right anterior insula tracked unbiased RPE only in participants with bipolar disorder [$M = .17$, $SD = .24$, $t(20) = 3.36$, $p = .00087$, $d = .72$], and not in matched controls [$M = .0042$, $SD = .18$, $t(20) = .11$, $p = .46$, $d = .024$].

Modulation of striatal connectivity by momentum

To further understand why only individuals with bipolar disorder exhibit momentum-biased striatal RPEs and whether this is driven by the mood momentum signals we identified in insula activation, we quantified the coupling between these two regions, and how it is affected by momentum of changes in reward i.e. we included model-estimated momentum values as a separate parametric regressor in functional connectivity analyses (see Supplementary Formula above for details on how model-estimated momentum values are computed).

Participants with bipolar disorder diverged from the control group in how their left anterior insular-striatal connectivity is modulated by perception of increased momentum [$t(40) = 3.14$, $p = .0016$, $d = .99$] with only matched controls showing a significant modulation [Control group: $t(20) = 3.58$, $p = .00046$, $d = .73$; Bipolar group: $t(20) = -1.04$, $p = .15$, $d = -.25$] (Figure S2).

Neither manic and depressive symptoms nor impulsive traits significantly predicted striatal-left insular functional connectivity modulated by periods of higher momentum (Table S3).

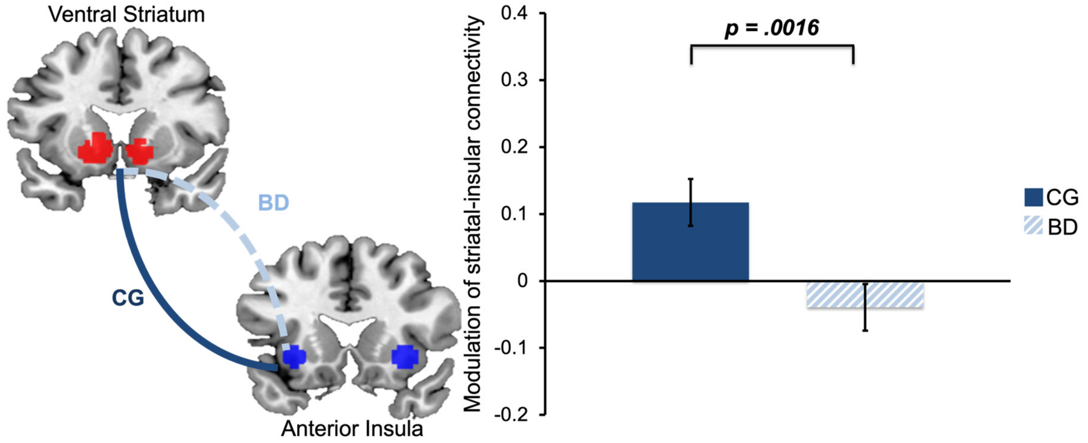

Figure S3. Stronger modulation of left anterior insular-ventral striatal functional connectivity by periods of higher momentum in matched controls than participants with bipolar disorder. Bilateral ventral striatum-left anterior insular functional connectivity modulated by periods of momentum during outcome was significantly different between groups (error bars: standard error of the mean). The solid line represents significant modulation in the control group (CG) and dashed line represents non-significant modulation in participants with bipolar disorder (BD).

Table S3. Linear regression coefficients with clinical symptoms and traits as predictors of left anterior insular-ventral striatal functional connectivity modulated by momentum-biased RPE in participants with bipolar disorder.

Predictor	*B*	95% CI for *B*	β	*t*	*p*	Tolerance	VIF
Intercept	-.38	[-.99, .24]		-1.30	.21		
MAS	-.01	[-.27, .25]	-.02	-.07	.94	.84	1.19
HAMD-17	-.01	[-.03, .02]	-.11	-.45	.66	.91	1.10
BIS-11	.01	[-.00, .01]	.30	1.21	.24	.91	1.11

R^2 = .088, *n* = 21, *F(3,20)* = .55, *p* = .66. MAS = Bech-Rafaelsen Mania Scale; HAMD-17 = Hamilton Depression Rating Scale; BIS-11 = Barratt Impulsiveness Scale; CI = confidence interval, VIF = Variance Inflation Factor.

4. Supplementary Discussion

The supplementary results reported here extend the main analyses by exploring the influence of model-estimated momentum values on left anterior insular-ventral striatal functional connectivity. These findings provide insight into how these neural processes are affected in periods of higher and lower momentum. Only matched controls, and not participants with bipolar disorder, increased striatal-insular coupling in response to higher momentum of recent outcomes. This corroborates our main findings of stronger striatal-insular functional connectivity in matched controls compared to participants with bipolar disorder as momentum-biased RPEs become more positive, which occurs primarily during upward momentum.

As we did not find that ventral striatal activity tracked momentum-biased RPE in matched controls in our main findings, we reason that the significant positive modulation of striatal-insular connectivity by momentum-biased RPE could arise from changes in the momentum signal: i.e. instances where momentum-biased RPE deviates from zero correspond to instances when momentum similarly becomes strongly positive or negative. We speculate that greater ventral striatal coupling with the anterior insula helps to contextualise reward perception and reduces the chances of misperceiving the likelihood of getting future rewards from the environment. The lack of contextualisation via the striatal-insular coupling in participants with bipolar disorder could therefore result in a greater propensity to misperceive cues in the environment when mood is strongly positive or elevated, which could result in recursive cycles where expectations of reward, moods and behaviours escalate to extremes.

5. Supplementary References

1. Eldar E, Niv Y (2015): Interaction between emotional state and learning underlies mood instability. *Nature Communications*. 6:6149.
2. Eckblad M, Chapman LJ (1986): Development and validation of a scale for hypomanic personality. *Journal of Abnormal Psychology*. 95:214-222.
3. Faul F, Erdfelder E, Lang AG, Buchner A (2007): G*Power 3: a flexible statistical power analysis program for the social, behavioral, and biomedical sciences. *Behav Res Methods*. 39:175-191.
4. Vinckier F, Rigoux L, Oudiette D, Pessiglione M (2018): Neuro-computational account of how mood fluctuations arise and affect decision making. *Nature Communications*. 9:1708.
5. Rutledge RB, Moutoussis M, Smittenaar P, Zeidman P, Taylor T, Hrynkiewicz L, et al. (2017): Association of Neural and Emotional Impacts of Reward Prediction Errors With Major Depression. *JAMA Psychiatry*. 74:790-797.
6. Rutledge RB, Skandali N, Dayan P, Dolan RJ (2014): A computational and neural model of momentary subjective well-being. *Proceedings of the National Academy of Sciences*. 111:12252-12257.
7. Wilson RC, Niv Y (2015): Is Model Fitting Necessary for Model-Based fMRI? *PLOS Computational Biology*. 11:e1004237.
